# Bone morphogenetic protein‐2 incorporated calcium phosphate graft promotes peri‐implant bone defect healing in dogs: A pilot study

**DOI:** 10.1002/cre2.613

**Published:** 2022-07-07

**Authors:** Henri J. J. Uijlenbroek, Xingnan Lin, Tie Liu, Yuanna Zheng, Daniel Wismeijer, Yuelian Liu

**Affiliations:** ^1^ Department of Oral Cell Biology Academic Centre for Dentistry Amsterdam (ACTA) Amsterdam The Netherlands; ^2^ School/Hospital of Stomatology Zhejiang Chinese Medical University Hangzhou Zhejiang China; ^3^ Department of Oral Implantology, the Affiliated Hospital of Stomatology, School of Stomatology Zhejiang University School of Medicine Hangzhou Zhejiang China; ^4^ Private Practice prof. dr. D. Wismeijer Ellecom The Netherlands

**Keywords:** biomimetics, BMP‐2, bone morphogenetic proteins, calcium phosphate, dogs, peri‐implantitis

## Abstract

**Objectives:**

The evaluation of three different drug delivery modes of bone morphogenetic protein‐2 (BMP‐2) in healing peri‐implant bone defects in beagle dogs. BMP‐2 was incorporated in or onto calcium phosphate (CaP) granules in various ways: (i) directly on the outer layer of granules CaP: as an adsorbed depot; (ii) during the entire precipitation process of CaP: an internally incorporated depot; or (iii) during the biomimetic coating precipitation of BMP‐2 on the surface of CaP granules: as a coating incorporated depot.

**Material and Methods:**

After extraction of the lower molars and wound healing in 6 male beagle dogs, 36 implants were placed (*n* = 6 animal per group). Peri‐implant bone defects were induced. The following treatment groups were evaluated: no treatment; air abrasive surface cleaning (SC) using hydroxyapatite; SC and the subsequent filling of the defect with CaP without BMP‐2; SC plus the subsequent filling of the defect with CaP adsorbed BMP‐2; SC plus the subsequent filling of the defect with CaP internally incorporated BMP‐2; SC plus the subsequent filling of the defect with CaP coating incorporated BMP‐2. Histological and histomorphometric analyses were carried out to quantify and compare the changes in bone tissue surrounding the treated implants.

**Results:**

In Group 1 with no treatment, four implants were lost. Group 5 with the SC and the subsequent filling of the defect with internally incorporated BMP‐2 biomimetically prepared CaP (BioCaP), whereby the BMP‐2 is incorporated in the entire volume of all BioCaP particles, showed overall the best results to regenerate bone around the implants.

**Conclusion:**

This study concluded that the group treated with SC plus the subsequent filling of the defect with CaP BMP‐2 internally incorporated BMP‐2, whereby BMP‐2 has been incorporated in the entire volume of all CaP particles, showed overall the best results when aiming to regenerate bone around the implants.

## 1 INTRODUCTION

Replacing missing teeth with dental implants has become a common treatment (Cairo et al., 2018). It is reliable and predictable and high implant survival rates have been reported (Derks et al., 2015; van Velzen et al., 2015). Peri‐implant health is characterized by the absence of erythema, bleeding on probing, swelling, and suppuration (Berglundh et al., 2018). Unfortunately, inflammation of the tissues surrounding the implant does occur (Derks et al., 2015). Peri‐implantitis has been defined as a plaque‐associated pathological condition occurring in tissues around dental implants, characterized by inflammation in the peri‐implant mucosa and subsequent progressive loss of supporting bone. The definition and prevalence of peri‐implant mucositis and peri‐implantitis differs in studies. Peri‐implantitis, defined as bleeding on probing with a probing depth of 4 mm, is reported in 34% of the patients and 11% of the implants (Giraldo et al., 2018). Peri‐implant mucositis occurs in about 80% of the patients and 50% of the implants (Lindhe et al., 2008). In a 10‐year prospective cohort study, defining peri‐implantitis as bleeding on probing with advanced bone loss ≥1.5 mm after loading, peri‐implantitis is reported in 7% of the dental implants (van Velzen et al., 2015) up to 14.8% of the patients. Severe peri‐implantitis will lead to implant loss, which makes its prevention highly important (Jepsen et al., 2015). If this fails, and peri‐implantitis does arise, treatment is indicated. Numerous treatment protocols that are clinically effective have been reported. However, no protocol has proven to lead to predictable treatment results (Heitz‐Mayfield & Mombelli, 2014; Roccuzzo et al., 2018; Smeets et al., 2014). The consensus is that surgical intervention is indispensable in the case of severe peri‐implantitis (Lindhe et al., 2008; Smeets et al., 2014). Debriding and decontaminating the implant surface and filling the defect with regenerative material can lead to the resolution of the inflammatory lesion (Lindhe et al., 2008).

There is evidence that plaque and biofilm are the principal etiological factors in peri‐implantitis defects (Berglundh et al., 2018). The International Union of Pure and Applied Chemistry defines biofilm as: “An aggregate of microorganisms in which cells that are frequently embedded within a self‐produced matrix of extracellular polymeric substances (EPSs) adhere to each other and/or to a surface” (Belibasakis et al., 2015; Vert et al., 2012). Removing the biofilm by cleaning the implant surface reduces the number of microorganisms associated with peri‐implantitis. In vitro experiments show air abrasive cleaning to be effective on machined, sandblasted large‐grit acid‐etched and titanium plasma‐sprayed surfaces (Louropoulou et al., 2014). It is concluded that the air powder abrasive with sodium bicarbonate powder has the smallest impact on the fibroblast–titanium surface interaction after treatment of smooth or structured titanium surfaces, even in the presence of plaque contamination (Louropoulou et al., 2015).

A characteristic of peri‐implantitis is the peri‐implant bone defect, which makes regenerative procedures a possible treatment option. Autologous bone, allografts, xenografts, and different biomaterials can all be used for bone regeneration. However, there is no consensus on augmentation therapy with a predictable outcome (Heitz‐Mayfield & Mombelli, 2014; Smeets et al., 2014). The results of the use of autologous bone and bone regeneration material are similar (Figuero et al., 2014; Smeets et al., 2014). Xenograft materials show better results when used in combination with resorbable membranes than without (Smeets et al., 2014). Biomaterials try to mimic nature, but not all deliver messenger agents. An osteoconductive biomaterial only enhances the migration of cells. To heal a critical‐sized bone defect—a bone defect that is too large to heal spontaneously within a lifetime—osteoinduction is required (Albrektsson & Johansson, 2001). Therefore, bone regeneration materials should preferably be osteoconductive and osteoinductive. Previously an animal model has been developed whereby stainless‐steel orthodontic ligatures were used in beagle dogs to create peri‐implant bone defects (Lin et al., 2017).

A common osteoinductive agent is bone morphogenetic protein‐2 (BMP‐2), a member of the family of transforming growth factors. It is a disulfide‐linked dimeric protein molecule with two major subunit species of 114 and 131 amino acids and stimulates the differentiation of mesenchymal stem cells into osteoblasts. It has been approved by the Food and Drug Administration, has been clinically used since 2007, and has been shown to induce bone formation (Lin et al., 2015; Y. Liu et al., 2010). For BMP‐2 to be applied in vivo, a carrier or scaffold has to be used. A common procedure is to functionalize collagen sponges by the adsorption of several milligrams of BMP‐2 (e.g., INFUSE®), aimed at promoting the healing of critical‐sized bone defects. BMP‐2 is released rapidly in a single high‐dose burst (Wang et al., 2014). This clinical use of BMP‐2 is associated with a number of serious adverse effects, which have been explained by the rapid release (associated with overdosing) of BMP‐2 into the surrounding tissue (Y. Liu et al., 2018). The effectiveness of BMP‐2 depends on its release kinetics (Y. Liu et al., 2007). Therefore, a contained controlled‐release system delivering BMP‐2 in a long continuous low dose is considered necessary. Using BMP‐2 when treating peri‐implantitis bone defects in animal models has been reported to increase bone formation around the implant (Sun et al., 2012; Xu et al., 2016).

Human bone consists of about 60% calcium phosphate (CaP). Therefore, CaP is often used in bone augmentation procedures (Yamada & Egusa, 2018). CaP can also be used as a carrier of osteoinductive agents. CaP itself has mainly osteoconductive properties. Proteins, such as BMP‐2, can be incorporated into CaP using a biomimetic coating technique (Lin et al., 2015), after which, under cell‐mediated CaP degradation, a local, slow, and sustained release of BMP‐2 will occur (Y. Liu et al., 2018), thus creating osteoinductivity.

The protocols for the incorporation of BMP‐2 into CaP, creating biomimetically prepared CaP (BioCaP), are well documented (Lin et al., 2015; Y. Liu et al., 2010, 2018). BioCaP coatings can be realized with the nucleation and formation of bone‐like crystals on a pretreated substratum (Lin et al., 2015). Starting with a thin seeding layer of amorphous CaP (ACaP), crystalline layers of octa CaP (OCP) are deposited on top of the seeding layer to form BioCaP granules. BMP‐2 can be coprecipitated into the latticework of the crystalline OCP layers. The release kinetics of BMP‐2 depend on the way in which it has been incorporated in or onto the carrier material (Y.Liu et al., 2018). Several approaches when incorporating BMP‐2 in or onto BioCaP are possible, resulting in different BMP‐2 concentration depots. By directly adding a BMP‐2‐containing solution onto the pre‐existing BioCaP granules, BMP‐2 will be adsorbed, (i) a surface adsorbed depot (Y. Liu et al., 2007). When BMP‐2 is added to the supersaturated CaP solution during the formation of the crystalline layers, during the formation of BioCaP granules, BMP‐2 will be incorporated into BioCaP: (ii) an internally incorporated (ins) depot (Y. Liu et al., 2007). By placing BioCaP granules in a supersaturated CaP solution with BMP‐2, new crystalline layers with incorporated BMP‐2 will precipitate on the existing BioCaP granules: (iii) a coating incorporated (inc) depot (Y. Liu et al., 2007).

The aim of this study is to determine the most effective healing of peri‐implant bone defects with BMP‐2 adsorbed and incorporated in or onto BioCaP, in an animal model. Three different modalities have been used: (i) BioCaP with a BMP‐2 adsorbed (ads) depot; (ii) BioCaP with a BMP‐2 internally incorporated (ins) depot; (iii) BioCaP with a BMP‐2 coating incorporated (inc) depot.

## 2 MATERIALS AND METHODS

### 2.1 Study design

The experiment was approved by the committee of the Board of Animal Experiments, traditional Chinese Medicine University (Hangzhou, China), according to Chinese law (ZSLL‐2011‐65).

**Figure 1:**
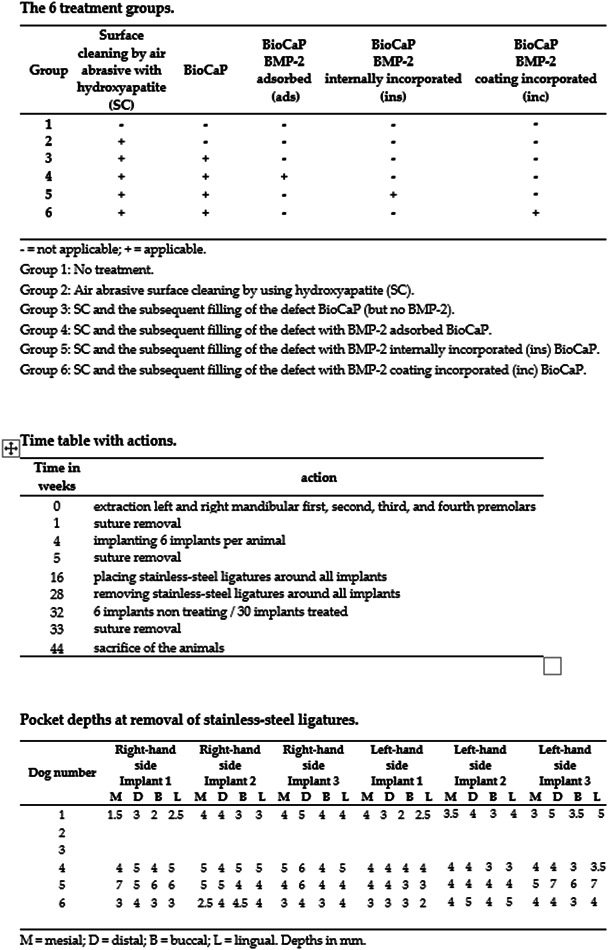
Top: Table displaying the six treatment groups. Middle: Timetable with actions. Bottom: Pocket depths at the removal of stainless‐steel ligatures. BioCaP, BMP‐2, bone morphogenetic protein‐2.

Six healthy 2‐year‐old male beagle dogs weighing between 13 and 14 kg were randomly selected. Extractions of left and right mandibular first, second, third, and fourth premolars were carried out. After 4 weeks, six implants were placed in each dog and numbered from one to six. A peri‐implant bone defect was created in all dogs, after which each implant in each dog was treated following a different treatment procedure as described in Groups 1–6. Random allocation was performed by numbering the dogs in any order from 1 to 6. Each treatment procedure was allocated to the implant locations by rotation. Treatment procedure 1 was started in dog 1 at the right most distal implant, procedure 2 at the second to last right implant and so on, so treatment procedure 6 ended in dog 1 at the left‐most distal implant. In dog 2, treatment procedures 1 to 6 started at the second to last right implant so that the treatment procedure 6 was performed on the right‐most distal implant. In dog 3, treatment procedure 1 started at the third to last right implant, and so on. Thus, each group of treated implants are in a different location in each dog. The six treatment groups are displayed in Figure 1.

### 2.2 Material preparation

Hydroxyapatite powder consists of hydroxyapatite rounded particles of 5–35 µm (CAM Bioceramics, Leiden, Netherlands). Human recombinant BMP‐2 (INFUSE" Bone Graft, Medtronic, Minneapolis, MN, USA) was introduced into the CaP solution or the coating solution at a concentration of 1 µg/ml. Samples of BioCaP but without BMP‐2; with BMP‐2 adsorbed (ads); BMP‐2 internally incorporated (ins); and BMP‐2 coating incorporated (inc) were prepared using the biomimetic mineralization approach as described in previous articles (Y. Liu et al., 2010, 2018; Uijlenbroek et al., 2021). Our procedure has been extensively described in a previous publication (Y. Liu et al., 2010) and is explained shortly in the following paragraph.

The biomaterial was immersed in a fivefold concentrated simulated body fluid for 24 h at 37°C, while stirring at 150 r.p.m. The fine, dense layer of ACaP formed this way served as a seeding substratum for the deposition of a more substantial crystalline layer. After freeze‐drying of the obtained material, the crystalline layer was produced by immersing the ACaP biomaterials in a supersaturated CaP solution at 37°C for 48 h, while shaking at 60 r.p.m. The samples were then freeze‐dried. The amount of incorporated BMP‐2 was determined using the enzyme‐linked immunosorbent assay (ELISA) technique (Wu et al., 2011). About 35 µg of BMP‐2 was eventually incorporated into each sample of 0.22 g BioCaP granules of Groups 5 and 6, and 35 µg of BMP‐2 was adsorbed to each sample of 0.22 g BioCaP granules of Group 4.

### 2.3 Surgical procedures

Every surgical intervention was carried out under general anesthesia by administering pentobarbital sodium (25 mg/kg) with the addition of penicillium (50 mg/kg) and atropine (0.03 mg/kg) at 30 min before surgery. Additional local anesthesia (1% lidocaine with 1:100,000 adrenaline) and skin disinfection (0.5% iodophor solution) was performed immediately before surgery. The intervention timeline is described in Figure 1.

In each beagle dog, eight premolars were extracted: the left and right mandibular first, second, third, and fourth premolars using tooth elevator and dental forceps, after which the extraction wounds were sutured. After 4 weeks of healing, an incision was made through the mucosa to the crest of the alveolar ridge. Buccal and lingual flaps were elevated. In each dog on each side, three Straumann implants were placed with a length of 8 mm and a diameter of 3.3 mm (Straumann AG, Basel, Switzerland; art. number 033.501). The implants were placed in such a way that the fixture margin coincided with the bone crest. Straumann (Straumann AG, Basel, Switzerland; art. number 048.371) closure screws were placed on all implants. The flap was sutured, with the implants nonsubmerged, to allow the implant sites to heal and facilitate osseointegration. The sutures were removed after 7 days. After 12 weeks of healing, clinical (solid percussion sound, pockets < 2 mm) and radiological examinations (bone to implant contact [BIC] to the implant neck) indicated that all implants were stable and were osseointegrated. During these 12 weeks, professional cleaning of the implants was performed twice a week by brushing with a toothbrush. The clinical examination was performed by an experienced dentist using a periodontal probe (PCP 8 Hu‐Friedy Co., Chicago, IL, USA). The radiological examinations were carried out using standardized radiograph holders (Kodak 2100, Intraoral X‐ray System, Carestream Health, Inc., Croissy‐Beaubourg, France) with a digital imaging system. An experienced dentist judged the X‐rays.

**Figure 2:**
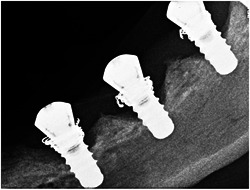
X‐ray before removing ligature with the bone defects of dog 6 left‐hand side

**Figure 3:**
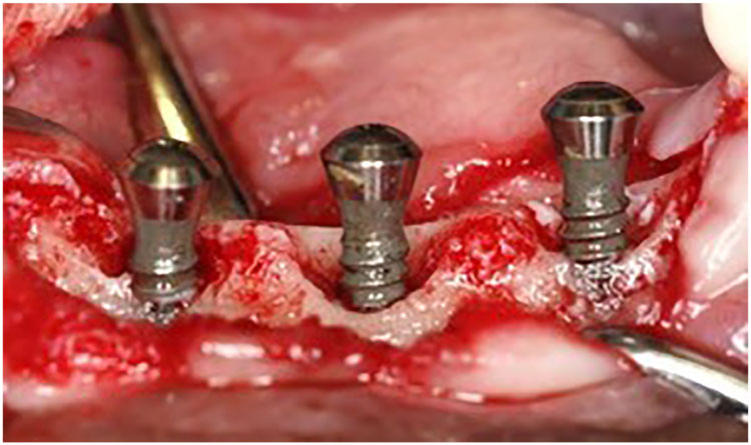
After removal of the peri‐implant granulation tissue by surface cleaning (SC) using an air abrasive technique combined with hydroxyapatite (SC); the induced peri‐implant bone defects are clearly visible.

With the aim of inducing peri‐implantitis resulting in peri‐implant bone defects, stainless‐steel ligatures (ligature wires, 0.010 in., 270‐0010, Ormco Cooperation, Brea, CA, USA) were placed in a submarginal position around the neck of the implants (Lin et al., 2017). The stainless‐steel ligatures were coiled around every implant six times for stability and were forced into an apical position on the peri‐implant mucosa. The excessive parts of the ligatures were cut off and the remaining ends were adjusted so as to avoid irritating the gingiva directly. When the ligatures were in place, the dogs were fed a soft diet. No cleaning of the implants was performed. The intended bone defects around the implants (Figures 2 and 3) were evaluated 12 weeks after ligation.

The stainless‐steel ligatures were then removed by cutting each coil and removing them. The dogs had a resting period of 4 weeks in which the tissues could adapt to the new situation. Then, the treatment was started and carried out according to the above‐mentioned treatment methods per group (Figure 1).

All bone defects were covered with a Bio‐Gide® membrane (Geistlich Pharma AG, Wolhusen, Switzerland). The soft tissue was sutured in place and the sutures were removed 7 days later. Immediately after surgery, penicillium (50 mg/kg) was administered for 3 days. The dogs were fed a soft diet. The implants were cleaned twice a week with a toothbrush. At 12 weeks postsurgery, the dogs were killed by intramuscular injection of an overdose of Sumianxin II. The parts of the jaw containing implanted dental implants were harvested and immediately immersed into 10% neutrally buffered formaldehyde for fixation.

### 2.4 Histological preparation and cutting

The harvested jaws parts were cut and bone blocks containing the implants with its surrounding tissues were obtained. The bone blocks were dehydrated in serial steps in alcohol, xylol, and then embedded in methylmethacrylate (MMA, Technovit® 7200 VLC Exact®, Heraeus Kulzer, Wehrheim, Germany).

Each implant was cut in half at random across its longitudinal axis. Parallel cuts were made through the sliced implant. Starting from the most peripheral part of the implant as many slides as possible were made. The slides obtained were cut‐grinded (Primus Cut Grinder, Walter Messner GmbH, Oststeinbek, Germany) into sections of approximately 250–300 µm. Only those slides, including the whole implant body, were further reduced to approximately 35 µm and these were polished and stained with toluidine blue.

### 2.5 Histomorphometric analysis

With a light microscope (Zeiss AxioCam Mrc5, Oberkochen, Germany), photos were taken of the slides, which were then directly loaded to a computer. An experienced blinded examiner performed the histomorphometric analysis using automated image analysis software (AxioVision, SE64 Rel.4.9 Zeiss software, Oberkochen, Germany).

**Figure 4:**
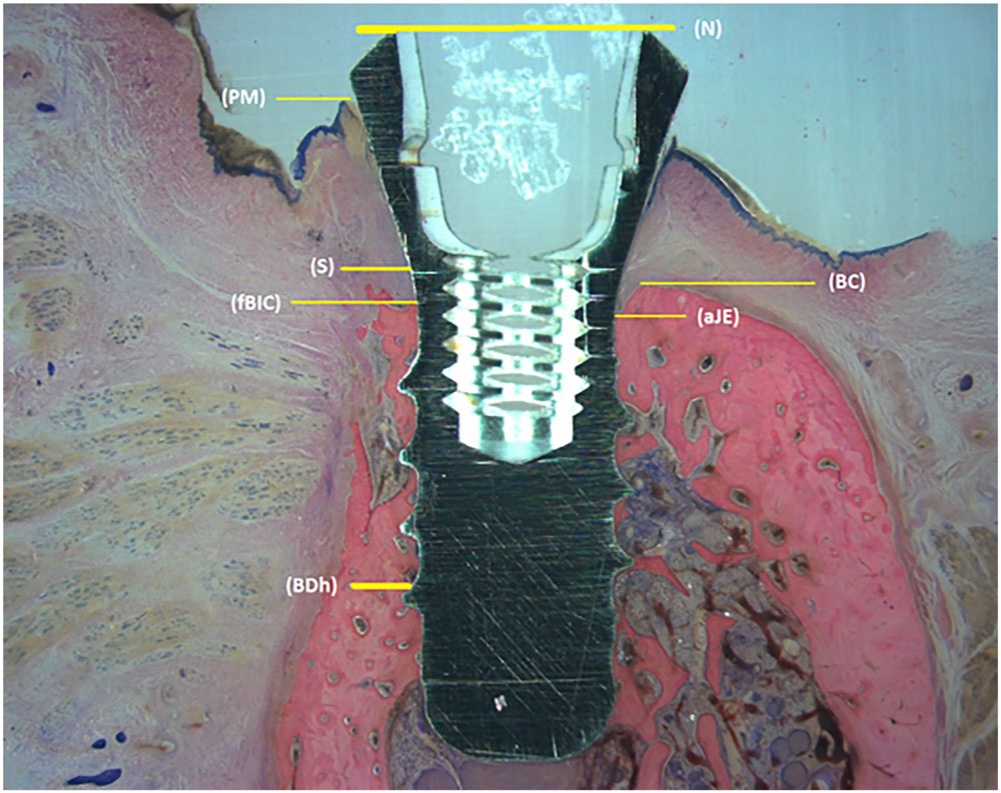
Schematic illustration of the landmarks used for histometric measurements

The following landmarks were identified in the stained sections (Figure 4):

N: The most superior part of the machined implant neck.

PM: The marginal position of peri‐implant mucosa.

S: The superior margin of the rough surface of the implant (a point not detectable in the specimens, but determined by hand after subtracting 2.8 mm from the top of the implant, which is the height of the machined implant neck in standard Straumann dental implants).

BC: The top of the adjacent bony crest.

fBIC: The first superior bone‐to‐implant contact.

aJE: The apical portion of the junctional epithelium.

BDh: The apex of the bone defect.

The regenerated areas, visible in high‐resolution magnification, were identified by the difference in color after staining. All measurements were calculated separately for the lingual and the buccal sides.

The measurements calculated by two examiners were:

BDh‐BC (mm): The vertical distance, parallel to the implant axis, between BDh and BC: the maximum possible augmentation height.

S‐BC (mm): The vertical distance, parallel to the implant axis, between S and BC: the crestal bone loss: the amount of bone loss after augmentation.

BIC (%): Bone‐to‐implant contact (BIC in %): the length of the implant where it is in contact with the newly formed bone in the regenerated area, as a percentage of the total rough surface length: the proportion of augmented bone.

BDh‐fBIC (mm): The vertical distance, parallel to the implant axis, between BDh and fBIC: the regenerated bone length: the amount of regenerated bone length in contact with the implant.

### 2.6 Statistical analysis

For each implant, the above‐mentioned measurements (BDh‐BC, S‐BC, BIC, and BDh‐fBIC) were calculated separately for each side. As we wanted to study the development around the entire implant, we considered the opposite sides together as one large group per implant. Mean values for the different variables were calculated for each implant. In order to compare the differences between the groups SPPS software (version 18 for windows, SPPS Inc., Chicago, IL, USA) was used, using the two‐way analysis of variance and the post hoc Bonferroni test for multiple comparisons. The null hypothesis—there is no effect of any regeneration procedure—was rejected at *p* < .05.

## 3 RESULTS

### 3.1 Material

As described in our previous articles (Y. Liu et al. 2005, 2010, 2018; Wu et al., 2010) the amount of encapsulated BMP‐2 was determined using an enzyme‐linked immunosorbent assay (Elisa, PreproTech EC, London, UK) showed that the amount of BMP‐2 encapsulated in the samples was approximately 35 µg of BMP‐2 per sample of 0.22 g of BioCaP.

### 3.2 Clinical results

After the extraction the wounds healed in all the dogs. All the implants were registered as integrated in Week 16 before the stainless‐steel ligatures were placed. In Week 19, 3 weeks after the placement of the stainless‐steel ligatures, large amounts of plaque appeared with the typical signs of perimucositis: erythema, bleeding on probing, swelling, and suppuration. Unfortunately, dogs 2 and 3 died between Weeks 16 and 28. At removal of the stainless‐steel ligatures in Week 28, no implant loss was experienced in the four remaining dogs. Clinical as well as radiological differences were observed between the implants in inflammation and pocket depths. A bone defect was present at every implant as illustrated in Figures 2 and 3. The amount of bone loss varied around the different implants. We used implants of 8 mm in length, whereas the pocket depths varied between 1.5 and 7 mm as shown in Figure 1. Four implants were lost between Weeks 28 and 32. There was no membrane exposure on killing in Week 44.

### 3.3 Histological findings

**Figure 5:**
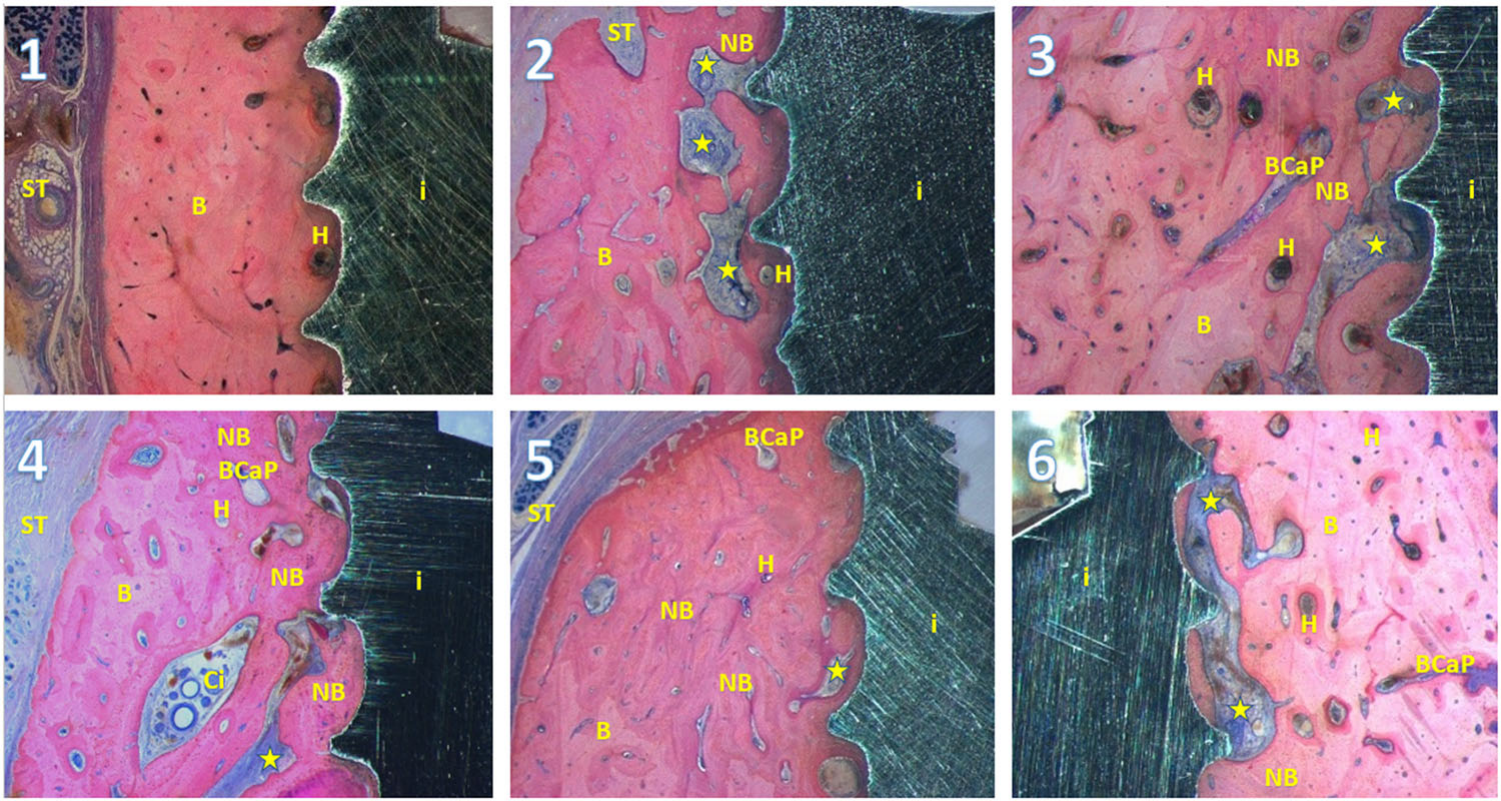
A histological image of each group. The numbers correspond with the treatment group. Asterix, hydroxyapatite; B, bone, BCaP, BioCaP; Ci, cell infiltrate of soft connective tissue with BioCaP; H, a Haversian canal; i, implant; NB, new bone; ST, soft tissue.

The difference between regenerated bone and “old” bone was visible with high magnification, as the color of the regenerated bone was more intense and slightly darker, which can be seen in Figure 4, coronal of BDh, and Figure 5. The regenerated bone was well adapted to the implant surface. In Figure 5, Groups 2, 3, 4, and 6 show a noninflammatory interconnection between the implant and the hydroxyapatite. Apparently, at surface cleaning of the implant with hydroxyapatite, some particles stick to the implant and remain there after 44 weeks. Between the hydroxyapatite particle and the implant, bone can be found (Groups 2, 3, and 6). Particles BioCaP can still be found after 44 weeks (Groups 3, 4, 5, and 6), although in these sections the smallest number of particles of BioCaP is seen in the BioCaP with internally incorporated BMP‐2. Looking at these sections, Groups 1 and 5 resemble each other the most. This can indicate the treatment with surface cleaning (SC+ BioCaP + BMP‐2) internally incorporated mimics natural bone the most. These images seem to show the biggest amount of new bone that can be found in the group SC + BioCaP + BMP‐2 internally incorporated.

### 3.4 Histomorphometric analysis

**Figure 6:**
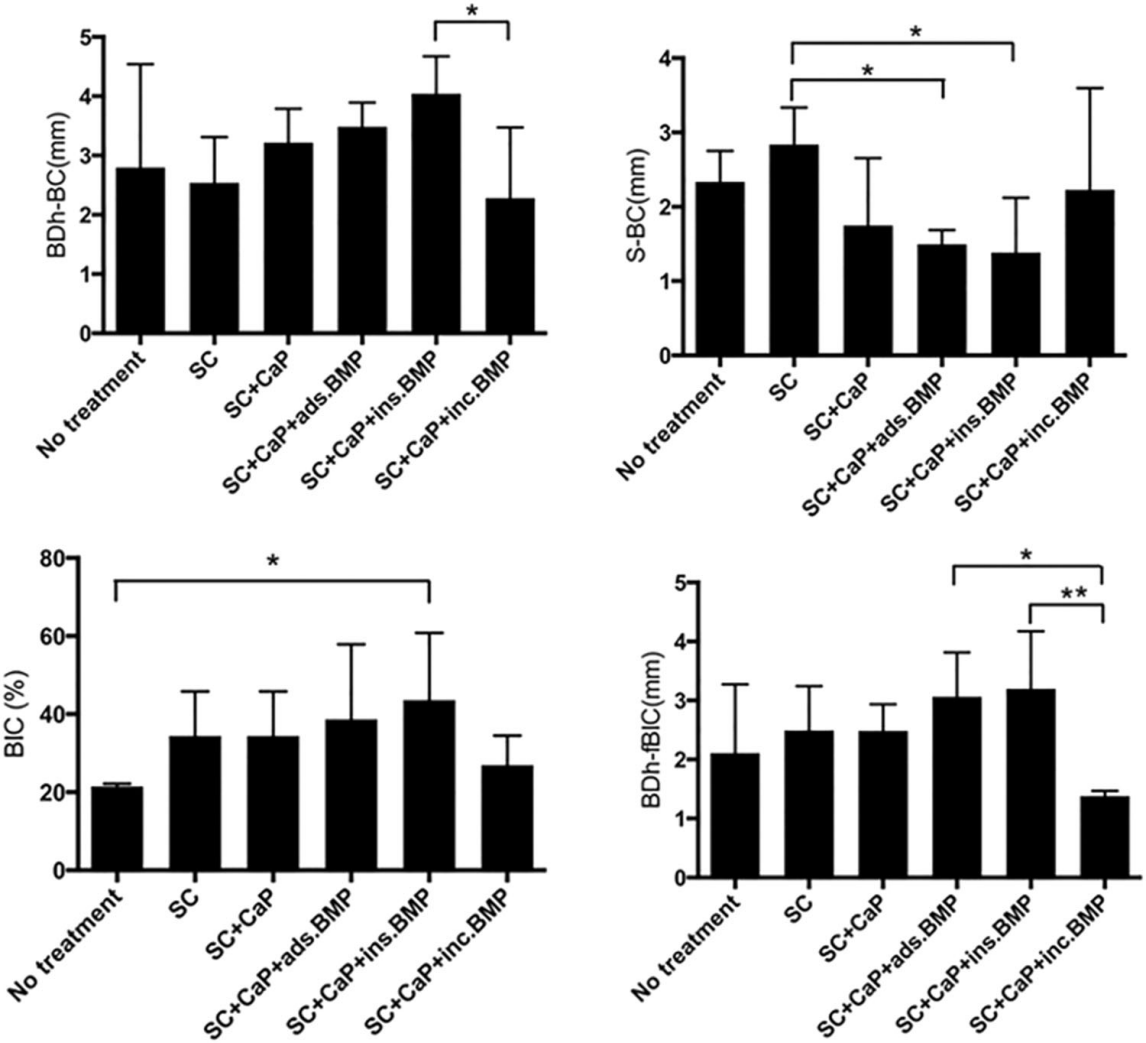
Statistical outcome of the different parameters. The asterix were mark where statistically relevant differences were found between the groups. ads, adsorbed; BMP, bone morphogenetic protein; CaP, calcium phosphate; inc, coating incorporated; internally ins, incorporated; SC, surface cleaning.

Statistically relevant differences for the maximum possible augmentation height (BDh‐BC in mm) are found only between Groups 5 and 6 (Figure 6).

Statistically relevant differences for crestal bone loss (S‐BC in mm) are found only between Groups 2 and 4 and between Groups 2 and 5 (Figure 6).

Statistically relevant differences for the BIC (in %) are found only between Groups 1 and 5 (Figure 6).

Statistically relevant differences in the amount of regenerated bone (BDh − fBIC, in mm) are found only between Groups 4 and 6 and between Groups 5 and 6 (Figure 6).

## 4 DISCUSSION

Peri‐implant mucositis precedes peri‐implantitis. How the conversion of the one stage into the other takes place is not completely understood (Schwarz et al., 2018). The case definition of peri‐implantitis differs in studies (Berglundh et al., 2018; Doornewaard et al., 2018), but there is agreement that the characteristics of peri‐implantitis are biofilm, bleeding on probing, suppuration, and bone loss around the implant (Berglundh et al., 2018). Treatment of peri‐implantitis has been reported with outcomes varying from 57% unsuccessful at 12 months (de Waal et al., 2016) to quite successful at 5 years (Heitz‐Mayfield & Mombelli, 2014). These various results are illustrative of the difficulty in properly defining and treating peri‐implantitis. There does not appear to be a relationship between probing depth or bleeding and mean bone loss (Doornewaard et al., 2018). The host response seems stronger with a biofilm adjacent to implants than with a biofilm adjacent to a natural tooth (Salvi et al., 2017). Experimental peri‐implantitis and periodontitis in mice with silk ligatures (Hiyari et al., 2018) showed a greater bone loss around the implants within 3 months compared to natural teeth, whereby 20% implant loss occurred and no natural teeth were lost. Tissue destruction seems to be more rapid and extensive in experimental peri‐implantitis, compared to clinical periodontitis (Salvi et al., 2017). Therefore, in both our previous (Lin et al., 2017) and in this animal model, it is to be expected that the experimental peri‐implant bone defects are larger than bone defects around natural teeth caused in the same time frame with the same biofilm. If it is possible in our animal model to treat larger peri‐implant bone defects successfully, it can be expected that smaller clinical bone defects can also be treated successfully. Therefore, this animal model seems a suitable model to study clinical bone regeneration and may add some clinically relevant information to treatment procedures.

However, various problems were encountered. Inducing peri‐implant bone defects in several beagle dogs will not have exactly the same effect on every dog, due to host variations. In this study, dog 5 showed the most progressive peri‐implant bone defects with deep pockets (see Figure 1). These deep pockets probably caused the loss of three implants in this animal. Some of the implants with deep pockets showed micromobility, perhaps preventing the origination of a stable blood clot required for bone regeneration (Wang & Boyapati, 2006). The micromobility of an implant will therefore possibly negatively influence the success of bone regeneration.

We consider severe peri‐implant defects as critical size bone defects because both will not heal spontaneously within a patient's lifetime. For these to heal, bone induction is necessary, which can be realized by the use of BMP‐2. A research model for the best delivery mode of BMP‐2 may be found by studying the healing of peri‐implant bone defects in beagle dogs.

The implants were placed on the rough surface just inside the bone, so at the time of implant insertion S‐BC is 0 mm. After the creation of peri‐implant bone defects, BC will be located more apically than at the time of implant insertion. A successful bone augmentation will bring BC closer to S. The shorter the distance S‐BC, the more coronal the bone crest is, which means more bone has been regenerated. So, the smallest bar in Figure 6 (S‐BC) displays the largest amount of bone regenerated. The bar of Group 5 is the smallest bar, as expected. But due to the loss of several implants, the statistics are poor and the outcome can only be an indication.

After treatment, there can still be a (less deep) bone defect, so the top of the bone crest may not be in contact with the implant (Figure 4). When looking at the maximum possible augmentation height as presented in Figure 6 (BDh‐BC), Group 5 shows the longest bar, indicating it has the largest possible augmentation height. Group 6 has the shortest bar here, creating the smallest possible augmentation height. Statistically relevant differences for the maximum possible augmentation height were found only between Groups 5 and 6. We lost a Group 6 implant in dog 5, which had a 7 mm pocket at the removal of the stainless‐steel ligatures. The loss of an implant with a potentially high BDh‐BC value will decrease the mean value of the group, which could justify the small maximum possible augmentation height of Group 6. In this (too) small sample size, it has a great effect on the statistics, thus making valid conclusions impossible.

Similar correlations are expected to be found in the ratio of the amount of augmented bone (Figure 6, BIC) and the amount of regenerated bone in contact with the implant (Figure 6, BDh‐BIC), as both indices show bone regeneration. The best results are shown with BMP‐2 internally incorporated (Group 5). In addition, Group 5 has the largest maximum possible augmentation height. The results in Group 1 are limited, which is to be expected as no treatment was applied and thus no bone regeneration was seen. In Figure 6, BDh‐fBIC Group 6 performs even worse than Group 1. A Group 6 implant was lost in dog 5, due to its very severe peri‐implant bone defect. Implants (Figure 1) of 7 and 8 mm were used in the pocket. The small remaining sample size probably has a high impact on this result. In addition, Group 6 also had the smallest maximum possible augmentation height and therefore the least amount of bone gain was to be expected. The results of Groups 2 and 3 are approximately the same. This could show that the application of BioCaP by itself does not promote bone formation. The results of Groups 4 and 5 are better and the BMP‐2 internally incorporated shows the best result in all cases. This could mean that the incorporation of BMP‐2 does promote bone formation and that the BMP‐2 internally incorporated shows the highest effect. The statistically relevant difference between the different groups must be interpreted with caution, as statistics are poor due to subject loss and thus reduced sample sizes.

BMP‐2 is a protein with a three‐dimensional shape determining its properties. By an external source, such as a change of the isoelectric point or heat, it will be denatured and lose its function. The carrier may therefore not influence the properties of BMP‐2. A superficially BMP‐2 adsorbed depot is released rapidly and exhausted within a few hours (Y. Liu et al., 2007). Our previous research (T. Liu et al., 2014) studying BioCaP with an internal and surface‐coated depot in vivo shows that newly formed bone was deposited directly on the BioCaP surface, and bone marrow was in close contact with BioCaP. The BMP‐2 internally incorporated seemed to have the best results. To liberate the BMP‐2 that is entrapped within the inorganic latticework the degradation of the coating is necessary. The larger the surface area, the faster the degradation, so BMP‐2 will be released at a higher rate. The main objective of this study is to investigate the best BMP‐2 delivery mode for treating peri‐implant bone defects. This could be caused by the ratio surface/incorporated BMP‐2, as we explain in the next paragraph.

We hypothesize that the amount of BMP‐2 incorporated in the coating incorporation procedure is the same as in the internal incorporation procedure. However, the method of incorporation influences the total amount of material. After three cycles of alternate immersion, the volume of BioCaP granules increases from the initial 5–20 μm to 250–1000 μm, including the crystalline outer layer (Y. Liu et al., 2017). Ten micrograms of BMP‐2 can be successfully incorporated into 0.07 cm^3^ of BioCaP (T. Liu et al., 2017). For the sake of simplicity, the granules are assumed to be spherical. The volume of a sphere with a radius *r* is determined by the formula: $\frac{4}{3}$ × *π* × (*r*)^3^. The volume of a sphere with radius *r* will increase after immersion to a sphere with radius *r* + *p*. The increase in volume after immersion is: $\frac{4}{3}\times \pi \times {\left(r+p\right)}^{3}-\frac{4}{3}\times \pi \times {\left(r\right)}^{3}$. The amount of BMP‐2 coating incorporated is the same as the amount of BMP‐2 internally incorporated if the volumes that are incorporated with BMP‐2 are identical. The formula is: $\frac{4}{3}$ × *π* × ${\left(r+p\right)}^{3}$ − $\frac{4}{3}$ × *π* × ${\left(r\right)}^{3}=\frac{4}{3}$ × *π* × ${\left(q\right)}^{3}$, whereby *q* is the radius of the granule BMP‐2 internally incorporated, *r* is the radius of the granule before coating, and *r* + *p* is the radius of the granule after BMP‐2 coating incorporation. The relationship between the radius of coating the granules incorporated versus internally incorporated can be described by: ${\left(r+p\right)}^{3}$ − *r* ^3^ = *q* ^3^.

When the same amount of BMP‐2 is incorporated, the granule size is larger in the coating incorporation procedure than in the internal incorporation procedure. A larger granule has a larger surface area. As a consequence, one could conclude that BMP‐2 is released more rapidly. This might explain the lower effectiveness of coating incorporation in this study and may also explain why in Figure 6 Group 5 showed the best results.

## 5 CONCLUSION

This study shows that 0.22 g BioCaP granules with a BMP‐2 depot of 35 µg can generate new bone formation around the peri‐implant bone defect. BioCaP with a BMP‐2 internally incorporated depot may be the most effective delivery mode. We hypothesize this may be caused by the particle size with the best ratio surface/incorporated BMP‐2 needed to incorporate the same amount of BMP‐2. This will need further research.

## AUTHOR CONTRIBUTIONS

**Henri J. J. Uijlenbroek**: Surgery; design; data analysis; writing; completing the manuscript. **Xingnan Lin**: Surgery; design, data analysis; and data collection. **Tie Liu**: Animal care; histological preparation. **Yuanna Zheng**: Data collection; histological preparation. **Daniel Wismeijer**: Manuscript writing; revision of the manuscript. **Yuelian Liu**: Funding; study design; animal care; data collection; finalizing the manuscript.

## CONFLICT OF INTEREST

The authors declare no conflict of interest.

## Data Availability

The data that support the findings of this study are available from the corresponding authors upon reasonable request.
